# Long Lived Photoexcitation Dynamics in *π*-Conjugated Polymer/PbS Quantum Dot Blended Films for Photovoltaic Application

**DOI:** 10.3390/polym9080352

**Published:** 2017-08-10

**Authors:** Ruizhi Wang, Xiaoliang Yan, Xiao Yang, Yuchen Wang, Heng Li, Chuanxiang Sheng

**Affiliations:** School of Electronic and Optical Engineering, Nanjing University of Science and Technology, Nanjing 210094, China; wangruizhi1986@gmail.com (R.W.); herryliang0930@njust.edu.cn (X.Yan); 310040612@njust.edu.cn (X.Yang); wangyuchennanjing@163.com (Y.W.)

**Keywords:** photoinduced absorption spectroscopy, organic inorganic hybrid materials, solar energy

## Abstract

We used continuous wave photoinduced absorption (PIA) spectroscopy to investigate long-lived polarons in a blend of PbS quantum dot and regio-regular poly (3-hexylthiophene) (RR-P3HT). The charge transfer from RR-P3HT to PbS as well as from PbS to RR-P3HT were observed after changing the capping ligand of PbS from a long chain molecular to a short one. Therefore, PbS could be used to extend the working spectral range in hybrid solar cells with a proper capping ligand. However, we found that the recombination mechanism in the millisecond time region is dominated by the trap/defects in blended films, while it improves to a bimolecular recombination partially after ligand exchange. Our results suggest that passivating traps of nanocrystals by improving surface ligands will be crucial for relevant solar cell applications.

## 1. Introduction

Bulk heterojunction (BHJ) of *π*-conjugated polymer (PCP) and quantum dots were widely used for organic-inorganic hybrid solar cells (HSCs) [1,2,3,4,5,6,7,8,9,10]. Low bandgap lead sulfide quantum dots (PbS QDs) are considered as a promising electron acceptor as a replacer of fullerene due to their tunable and broad absorption range, especially extending to the infrared region [11,12,13,14,15,16] and high electron mobility [17,18,19,20]. A recent study has shown that long alkyl chain ligands, such as the oleic acid (OLA) coating on the core of as-casted PbS QDs, impede efficient charge transfer in hybrid polymer/QD solar cells. This problem can be partially solved by changing the long chain ligand to a short one, resulting in a tremendous increase of the solar cell’s efficiency [21,22,23,24,25]. However, it is still much lower than its polymer-fullerene counterpart, with an efficiency of ~11% [22,26,27,28]. The sluggish development of HSCs with PbS quantum dots may be due to the complexity of organic-inorganic systems, as well as to the lack of knowledge about the mechanisms for charge transfer in hybrid polymer/QD systems, compared to polymer/fullerene blends.

In this work, we utilized continuous wave photoinduced absorption (PIA) spectroscopy with various intensities and temperatures as a function of modulation frequencies, to study ligand exchange effects on the optical properties, particularly on the dynamic of charge transfer and recombination in the millisecond time regime in regioregular poly(3-hexylthiophene) (RR-P3HT)/PbS QD blend films. Besides the well-known electron transfer from polymer to PbS QDs, we observed the hole transfer from PbS to P3HT as well. However, the recombination mechanism is basically trap-assisted recombination, as described by the Shockley, Read, and Hall (SRH) model [29]. This could be the main reason for limited solar cell efficiency.

## 2. Materials and Methods

All sample films were prepared under a nitrogen atmosphere in a glove box with moisture and oxygen levels less than 1 ppm. All reagents were purchased from Sigma-Aldrich (Sigma-Aldrich Shanghai Trading Co. Ltd., Shanghai, China). Quartz substrates were cleaned by ultrasonication for 30 min in acetone and ethanol, followed by UV ozone treatment for another 30 min. The OLA-capped PbS QDs were synthesized according to the method of Hines and Scholes [30]. Briefly, 5 mmol PbO and 5 mL OLA were mixed in 20 mL octadecene. The mixture was heated at 170 °C in a nitrogen atmosphere until all PbO was dissolved. Meanwhile, another 1 M hexamethyl disilathiane (HMD) in octadecene was prepared. The mixture solution temperature was lowered from 170 to 120 °C, and 2 mL of the 1 M solution of HMD was injected at 120 °C, causing the mixture to turn dark immediately. Then, the mixture was removed the heating plate immediately and cooled to room temperature. The resulting quantum dots were isolated by precipitation in acetone and re-dispersed in chloroform. The ligand OLA exchange to the ligand acetic acid (AA) procedure was followed according to the published protocol [31,32]. That is, homemade PbS quantum dots and purchased RR-P3HT from Sigma-Aldrich (Shanghai, China) were mixed with a ratio of 1:2 by weight. The blend films were then immersed into 0.01 M acetic acid solution in acetonitrile for 30 min under continuous stirring. Finally, the film was washed with excess acetonitrile and dried at 110 °C for 15 min [33].

The steady-state photo modulated (PM) spectrum was obtained using a standard continuous wave setup [34,35]. We used a diode laser at *ћ*ω*_L_* = 1.54 eV (*λ* = 808 nm) and *ћω_L_* = 2.77 eV (*λ* = 447 nm) for excitations. An incandescent tungsten/halogen lamp was used as the probe beam. The pump and probe beams were overlapped on the samples with a 1-mm radius spot in a cryostat, in which the sample temperature could vary from 77 to 300 K. After, the transmitted probe beam was detected by a silicon detector through a monochromator. The changed transmitted probe beam (Δ*T*), which was caused by the modulated pump beam, was detected by the phase-sensitive technique. For the modulation-frequency dependence measurement at a fixed probe wavelength, the pump beam was modulated from 5 Hz to 30 k Hz; the data were corrected by normalizing to the system response. An additional band pass filter (700 ± 5 nm) for delocalized polaron (DP) band or a long pass filter (>900 nm) for localized polaron (LP) bands was put before the sample to minimize additional influences from probe beam absorption, especially for low modulation frequencies [36,37].

## 3. Results and Discussions

**Figure 1:**
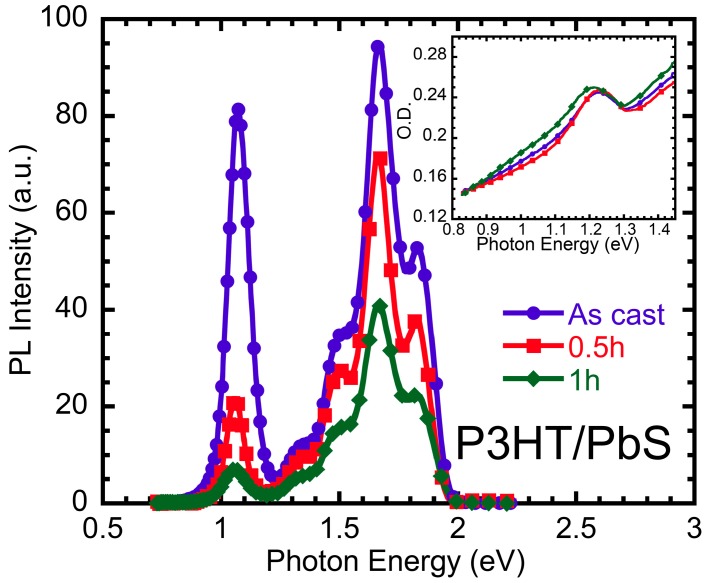
Photoluminescence spectra of RR-P3HT and PbS QD blend film in OLA-capped ligand (blue), AA-capped ligand exchanging process for 0.5 (red) and 1 h (green). The inset shows its normalized absorption spectra.

Figure 1 shows the absorption and photoluminescence (PL) spectra of RR-P3HT/PbS QD thin films in OLA-capped ligand (as-cast) and AA-capped ligand with the exchanging process for 30 min (0.5 h) and 1 h, respectively. The absorption range of RR-P3HT/PbS QD film is roughly between 1 and 1.55 eV, and the peak observed at 1.25 eV is related to the excitonic transition of PbS QDs [31]. Meanwhile, PL intensities of films decrease dramatically with the increasing time of the ligand exchanging process. The PL in 1.7 eV originated from P3HT and 1.1 eV originated from QDs ratio also increases. This is ascribed to the fact that the shorter ligand will facilitate more charge transfer under the interface of polymers and QDs, resulting in PL quenching [32] in both polymers and QDs as well as in the improvement of the device’s performance [38,39,40]. Similar PL quenching by charge transfer between the polymer and acceptor has been extensively observed in organic solar cell materials, being one way of showing the efficiency of charge generation [41].

**Figure 2:**
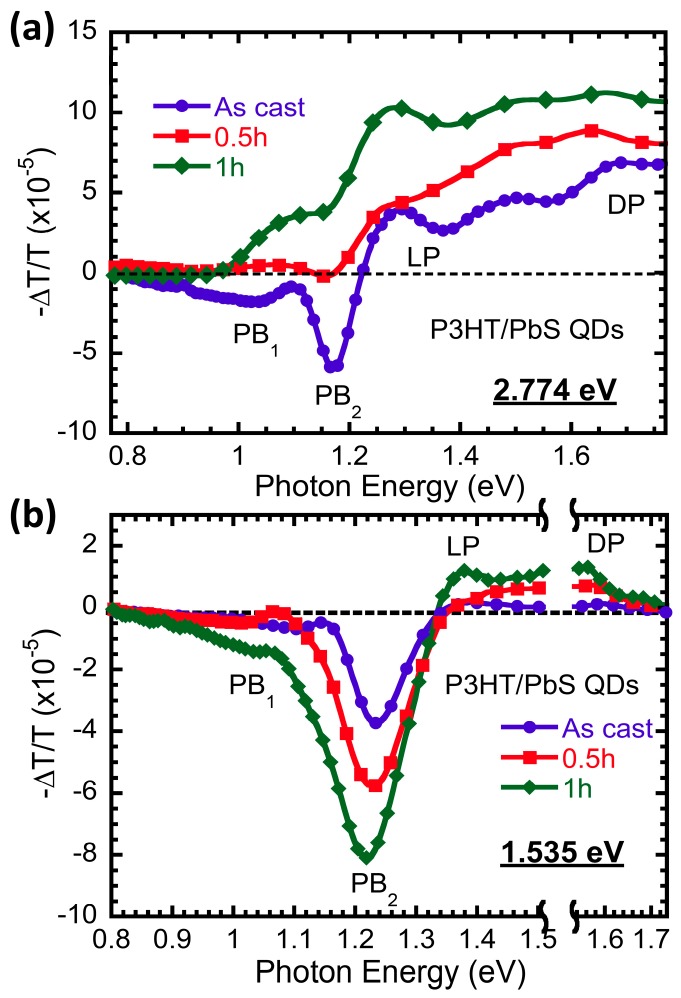
In-phase component of PIA spectra excited by 2.774 eV (**a**) and 1.535 eV (**b**) in RR-P3HT and PbS QD blend film with the OLA-capped ligand (blue circle) and AA-capped ligand exchange process for 0.5 h (red square) and 1 h (green rhombus).

To study charge transfer between the polymer and PbS quantum dots in the millisecond time region, we measured the photoinduced absorption (PIA) spectra with different photon energies. Figure 2a shows the in-phase component of PIA spectra excited by a 2.77-eV laser. The blue dotted line represents the standard featured PIA curve of as-cast films. Due to the highly ordered 2D lamellae sheets generating a strong interchain interaction in P3HT, the photoinduced absorption (PIA) peaks at ~1.3 and ~1.65 eV are attributed to localized polarons (LPs) and delocalized polarons (DPs), respectively [34,42], while the photoinduced bleaching (PB) at 1.18 eV is caused by a local electric field originating from the trapped charges of QDs [43,44]. Here we should point out the PB band at 1.1 eV due to the ground state absorption of PbS quantum dots is relatively weak, because of the short life constant (order of nanosecond) of excitons in quantum dots [4] as well as the spectra overlapping with the PIA band from RR-P3HT [45]. However, in AA-capped PbS QDs after ligand exchange, PIA intensity rises and PB intensity drops. This is especially the case in the sample treated after 1 h; the PB signal completely vanishes, as it is submerged by longer-lived polarons in the polymer. Also, the disappearance of the PB band at 1.18 eV suggests a lower density of traps in QDs after ligand exchange. This confirms that the decrease of PL intensity shown in Figure 1 is due to charge transfer between the polymer and PbS.

Figure 2b exhibits the in-phase component of PIA spectra using a 1.54-eV laser, in which the excitation energy is below the bandgap of RR-P3HT. Therefore, the photoexcitations are generated only in PbS just after photon absorption. There are no PIA bands related with long-lived photoexcitations, namely, LP and DP bands in RR-P3HT, observed in “as-cast” film. On the contrary, after ligand treating for 0.5 and 1 h, LP and DP bands emerge as the evidence of charge transfer between PbS and RR-P3HT. At the same time, we notice that the PB bands increase as well, due to trapped charges in QDs around 1.2 eV. This is unlike the above gap exaction shown in Figure 2a, suggesting there are relatively more charges trapped in QDs with 1.54 eV excitation. It is shown clearly that the PB_2_ band redshifts with ligand exchange from OLA to AA. This may indicate the weaker electric field caused by trapped charges of QDs after ligand exchange [44]. Nevertheless, the existence of PA bands originating from polarons in RR-P3HT clearly indicates holes transfer from PbS QDs to P3HT after ligand exchange.

**Figure 3:**
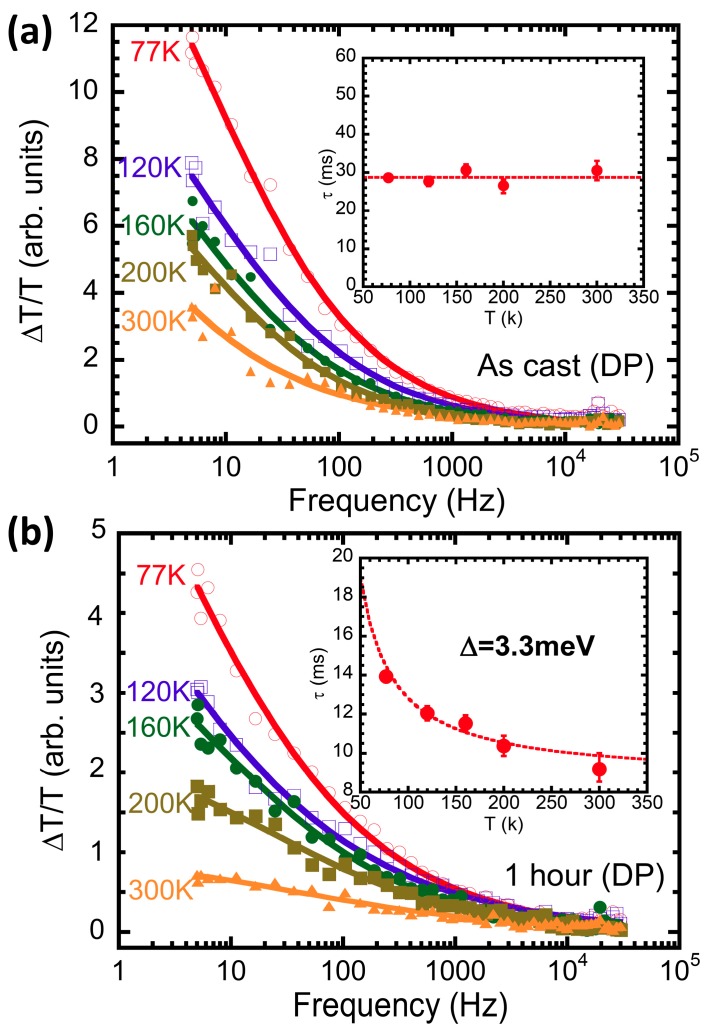
Modulation frequency dependence of PIA signals of DPs in RR-P3HT and PbS QD blend film with OLA-capped ligand (**a**) and AA-capped ligand exchange process for 1 h (**b**) at various temperatures. The insets of (**a**,**b**) are their lifetimes at various temperatures (Red dots are data and dash lines represent fitting curves).

To understand the recombination processes, we analyzed the PIA signal as a function of modulation frequency. The DP band was selected to minimize the influence of PB band of PbS. Photoexcitations with lifetimes longer than the inverse of the modulation frequency of the pump beam cannot fully recover within a pump’s cycle, thus the PIA signal will diminish as the modulation frequency increases, as shown in Figure 3a,b for polaron in blended films of “as-cast” and “1 h” ligand exchange, respectively, at various temperatures. Therefore, we obtained the average lifetime at various temperature, analyzed by fitting the total PIA signal, *R* = (in-phase^2^ + out-of-phase^2^)^0.5^, with a dispersive recombination equation [46]:

(1) $$-\frac{\Delta T}{T}=\frac{{\left(\frac{\Delta T}{T}\right)}_{0}}{1+{\left(\omega \tau \right)}^{\gamma }}$$

where (Δ*T*/*T*)_0_ is the steady-state response at 0 Hz in terms of the pump intensity, ω is the modulation frequency of the pump beam, τ is the averaging lifetime, and γ is the dispersive parameter which describes the dispersive degree of lifetime [47]. The Δ*T*/*T* scale versus the modulation frequency with different temperatures for DPs in P3HT/OLA-capped and P3HT/AA-1-capped PbS QD blend films excited at 35 mW/cm^2^ are shown in Figure 3a,b, respectively. The lifetime obtained from Equation (1) is shown in the respective inset and fitted by $\frac{1}{\tau }=\frac{1}{\tau_{0}}+\nu \exp\left(-\Delta /k_{B}T\right)$, where Δ is the activation energy, and ν and τ_0_ are parameters. The lifetime in the blend film before ligand exhange shows temperature independent, but temperature dependent with 3.3 meV thermal activated energy after ligand exchange. Therefore, the long-lived polaron in the millisecond time regime in the polymer is basically transported by tunneling [48]. Considering the lifetime in the blended film of 30 ms, we conclude that the energy barrier between the polymer and PbS is too high to be able to be thermally overcome [49]. Compared with the independent relation between τ and temperature in the as-cast sample, as shown in the inset of Figure 3a, it could be illustrated that short side chains cause the modification to the carrier recombination mechanism, i.e., they lower the barriers to make thermal activation possible. Thus, the thermal activation process can be observed in Figure 3b. However, the multistep tunneling process may still play a very important role, for the thermal activation energy of 3.3 meV is small compared to that (~160 meV) observed in MEH-PPV/PCBM blends [34].

**Figure 4:**
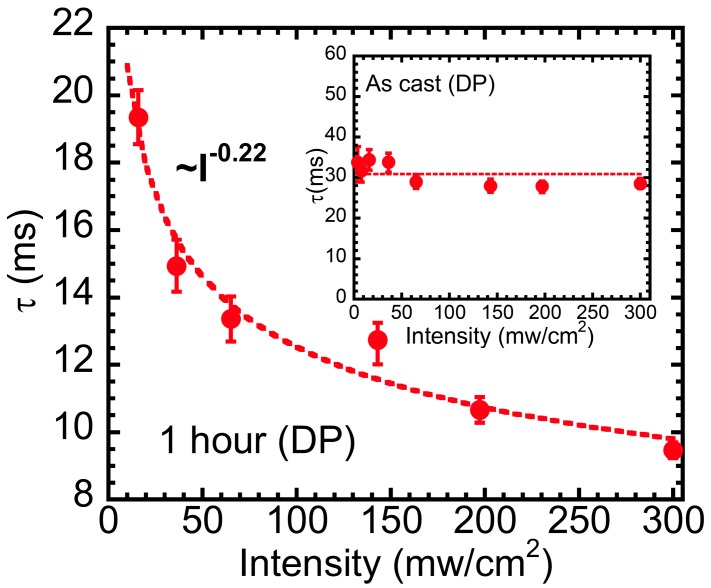
Lifetimes of DPs in RR-P3HT and PbS QD blend film with OLA-capped ligand (inset) and AA-capped ligand exchanging process for 1 h at various excitation intensities (Red dots are data and dash lines represent fitting curves).

To classify the recombination mechanism further, we measured the PIA signal as a function of excitation intensities at a fixed temperature. Ideally, the PIA scales linearly with I for monomolecular recombination (MR) kinetics, whereas it scales with $\sqrt{I}$ for bimolecular recombination (BR) kinetics at a quasi-steady state [50]. However, this simple approach is complicated by disorder, inhomogeneity, and impurities as well as traps. As it has been discussed in previous works [34,47], a more reliable method is to analyze the lifetime as a function of pump intensities. Lifetimes for DPs in film with and without ligand exchange measured at 77 K at various pump intensities are shown in Figure 4 and its inset, respectively. For film without ligand exchange, the lifetime is independent of excitation intensity as well; this is perhaps due to the recombination between polaron in P3HT and deep traps in PbS quantum dots, i.e., Shockley, Read, and Hall (SRH) recombination [29]. Since quantum dots have high surface-to-volume ratios, the surface trap states from unpassivated surface ions can intrinsically serve as recombination centers for charge carriers [51]. On the other hand, for ligand exchange films, the DPs lifetime has sublinear relation with excitation intensities (~$I^{-0.22}$), which however still is not suitable to the character of bimolecular recombination with Δ*T*/*T* reverse proportional to the square root of intensity (*I*^−0.5^) in this time regime. Therefore, we ascribed the recombination mechanism to be due to the joint effects of SRH and bimolecular recombination [50].

## 4. Conclusions

In conclusion, we observed the charge transfer from RR-P3HT to PbS quantum dots, as well as from PbS to RR-P3HT, in same the blended film after changing the capping ligand of PbS from a long chain molecular to a short one. Our work proves that PbS QDs can be used to extend the working spectral range in hybrid solar cells, and that a short side chain employed as a capping ligand for PbS in blend films will enhance the charge transfer between the polymer and QDs. Through analyzing the intensity and temperature dependence of the PIA signal, we found that the recombination mechanism in the millisecond time region is basically dominated by the deep trap/defect in quantum dots, i.e., SRH recombination. However, certain improvements can be made with partially bimolecular recombination after ligand change. Our results suggest that the reason that blends of PbS and widely-used conjugated polymers exhibit an unsatisfactory performance in bulk heterojunction devices compared to polymer/PCBM blends is that the traps/defects in PbS quantum dots dominate the free carriers’ generation, transportation, and recombination. Therefore, passivating traps of nanocrystals by improving surface ligands will be crucial for HSCs in the future.
